# New Instruments for the Management of Cochlear Implantation in an Individual with a Fracture of the Temporal Bone and Cochlear Ossification

**DOI:** 10.1155/2024/3946072

**Published:** 2024-09-03

**Authors:** Antonio Frisina, Francesco Seno, Gionata Conni

**Affiliations:** ^1^ ENT Complex Operative Unit-Western District AULSS8 Berica Hospital, Valdagno, Italy; ^2^ MED-EL Italian Local Unit, Via Dei Vanga 1B, Bolzano 39100, Italy

## Abstract

Cochlear implant surgery can be highly complex in cases where ossification of the internal ear has taken place. In this case report, we report the use of new technological instruments to optimise the surgical process of implantation. These were the combined use of a surgical approach extended by a subtotal petrosectomy, a pre-operative radiological study with the OTOPLAN software for choosing the most suitable electrode array, and a residual functionality test of the auditory nerve using the ANTS test electrode array prior to inserting the cochlear implant electrode array. These were used to successfully treat a case of total deafness caused by a fracture in the temporal bone complicated with ossification of the basal turn of the cochlea. These instruments ensured that the operation was performed with excellent results, reducing the risk of failure to a minimum in this complex case.

## 1. Introduction

Individuals who have suffered a temporal bone fracture, especially transversal fractures, can exhibit deep neurosensorial hypoacusis. This can be due to several causes, including trauma to the otic capsule with damage to the organ of Corti and stria vascularis, damage to the neuroepithelium with a loss of ciliated cells and the spiral ganglion, and bleeding in the cochlear duct leading to profound hearing loss or deafness [1–5]. A cochlear implant (CI) is currently the gold standard for auditory rehabilitation for patients with profound sensorineural hearing loss in cases where there is an intact, functional cochlear nerve, and implantable cochlea [5]. In temporal bone fracture cases, the CI operation can be considerably more challenging than in conventional cases, as they are often accompanied by complications such as ossification within the scala tympani or low functionality of the auditory nerve. Histopathological studies of temporal bone fractures show that, on average, only a third of ganglion cells survive [1, 6, 7]. The number of surviving ganglion cells seems to be an important factor in determining the success with a CI [1, 4], although some histopathological studies on temporal bones have shown a low survival rate of ganglion cells in CI users that have nevertheless achieved good speech understanding outcomes of CI [1]. It appears to be the case that even a few ganglion cells can effectively transduce electrical signals from the CI to the ascending auditory pathways [3, 8].

A frequent consequence of trauma is intracochlear ossification, usually in the basal turn of the scala tympani [2, 3, 9]. The layout of the fracture lines and the labyrinthine ossification can complicate the insertion of the CI electrode array [2, 4, 5, 10]; therefore, relying on images is extremely relevant to making proper surgical considerations. It is important to consider that high-resolution computed tomography (CT) may not show 22% of luminal obstructions, while high-density magnetic resonance imaging (MRI) is much more reliable in identifying cochlear patency, facilitating a more reliable differential analysis between intracochlear fluid, fibrosis, and new bones [2, 3, 9].

This case report documents the treatment of a patient with unilateral total deafness due to a temporal bone fracture. This case was complicated with ossification of the basal turn of the cochlea and facial paralysis on one side, as well as neurosensorial hypoacusis on the contralateral side. The side of the fracture was chosen for the CI. The procedure involved the use of two innovative instruments: (1) software for radiological imaging analysis to plan the surgery, especially with regard to the ossification of the basal turn, and (2) the use of a test electrode array to assess the functionality of the auditory nerve prior to insertion of the final electrode array of the CI. We recommend the use of these instruments as part of a potential protocol to improve outcomes in similar cases.

### 1.1. Overview of the Clinical Case Study

A patient was examined for total deafness on the right side and neurosensorial hypoacusis on the left side. The average magnitude of the hearing loss on the left side was 41 dB HL in the 0.5–4 kHz range. The patient reported a traumatic brain injury in May 2013 with a left parietal temporal hemorrhagic contusion, a right parietal temporal fracture, a transverse fracture in the right petrous bone which involved the basal turn of the cochlea and the vestibule, with total deafness and right facial paralysis.

In July 2013, the patient received a surgical intervention to decompress the transmastoid in the right facial nerve, which was performed at another clinic. In March 2014, the patient was scheduled to receive a CI on their left side at another clinic; however, during the procedure, ossification of the scala tympani was discovered. It was impossible to achieve cochlear patency, even after milling; therefore, this procedure was aborted.

## 2. Materials and Methods

Five years later, in April 2019, the patient presented at our clinic. With preoperative CT imaging, ossification of the basal turn involving both scala was observed. Contrast MRI showed a cochlea with a regular appearance and a symmetrical morphology when compared to the contralateral cochlea. In T2-weighted sequences, maintenance of the hyperintense signal consistent with the presence of liquid was observed. The scan showed that cochlear and facial nerves were present, as well as the two components of the vestibular nerve in the right internal auditory canal, which was also assessed with a 3D right-angled sequence on its major axis. It was decided to implant a CI on the right side after a subtotal petrosectomy.

### 2.1. Radiological Planning

To better plan the surgical intervention, and to choose the most suitable electrode array, the OTOPLAN radiological analysis software was used (developed by CASCINATION, Bern, Switzerland, in collaboration with MED-EL, Innsbruck, Austria). OTOPLAN can estimate cochlear duct length [11], which can facilitate the selection of the most suitable electrode array to achieve full insertion (up to two cochlear turns).

OTOPLAN also allows for the basal turn to be observed and to measure the extent of the ossification after the round window. This enabled an electrode array to be chosen such that the active stimulation area could be placed more apically, bypassing the ossified basal region of the cochlea, where stimulation may be less effective.

**Figure 1:**
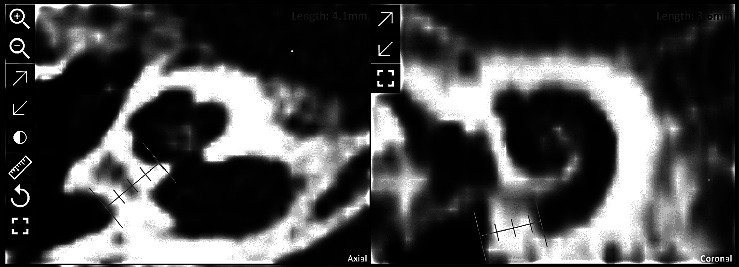
Radiologic analysis of the extent of the ossification in the basal turn of the cochlea.

The estimated implantable length was derived from the length of the scala tympani on its second turn (720°) at the organ of Corti, which was 31.7 mm. Ossification was measured to spread basally for 4 mm (see Figure 1).

### 2.2. Choosing the Electrode Array

**Figure 2:**
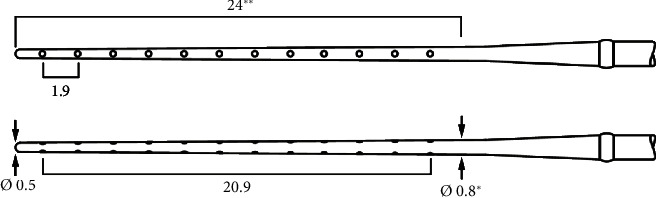
Schematic of the MEDIUM electrode array.

The electrode array chosen was the MEDIUM (MED-EL). It measures 31.5 mm in length in total, with an active zone of 24 mm, active stimulating range of 20.9 mm, and a passive zone of 7.5 mm (see Figure 2). Since an estimated 5 mm of drilling was necessary to open the cochlea, the aim was to place the array such that the passive 7.5 mm occupied this ossified area, which was potentially unresponsive to stimulation, and thus to concentrate the electrode contacts apically within the nonossified region of the cochlea.

### 2.3. Surgery

**Figure 3:**
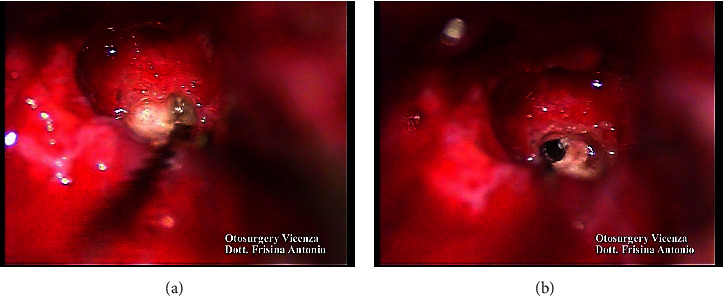
(a) Ossification of the scala tympani. (b) Open vestibular duct.

A subtotal petrosectomy approach was chosen in order to manage the middle ear access. Ossification of the basal turn was observed during the surgery. Patency of the vestibular duct was achieved via a cochleostomy and with drilling in the ossified area (Figures 3(a) and 3(b)).

### 2.4. Verification of the Functionality of the Auditory Nerve

**Figure 4:**
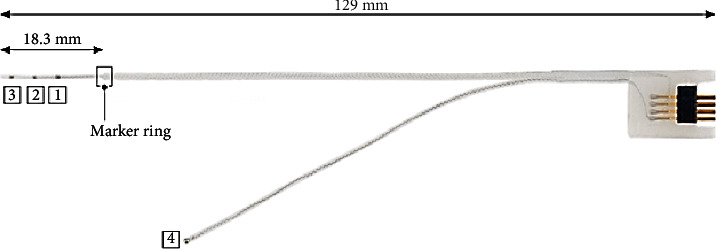
ANTS electrode array. The numbering shows the three intracochlear electrode contacts (1, 2, and 3) and the extracochlear electrode contact (4), which is placed under the temporal muscle.

Prior to electrode array insertion, residual functionality of the cochlear nerve was assessed via the auditory nerve test system (ANTS) test electrode (MED-EL) [12–14]. ANTS is composed of a stimulating intracochlear electrode array and a nonstimulating extracochlear ground electrode with a single contact, which is placed below the temporal muscle. The intracochlear array has three active contacts (numbered from 1 to 3) with an intracontact spacing of 0.4 mm, and a 0.8 mm spacing between the basal contact and the marker ring. The total length inserted into the cochlea is 18.3 mm (Figure 4).

**Figure 5:**
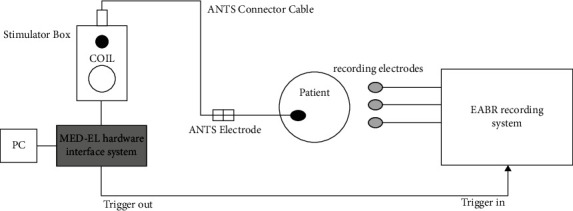
Schematic of use of the ANTS electrode array to evoke ABRs. Stimulation was carried out using a computer-controlled hardware interface (left side). The ABR recording was made using an evoked potential recording system (right side). The two systems were coordinated using a triggering system.

ANTS allows for the impedance of the electrode contacts to be measured, as well as for intracochlear stimulation to evoke auditory brainstem responses (ABR). The recording of eABRs was made by synchronising the ANTS interface with an ABR recording system using a trigger system (Figure 5).

**Figure 6:**
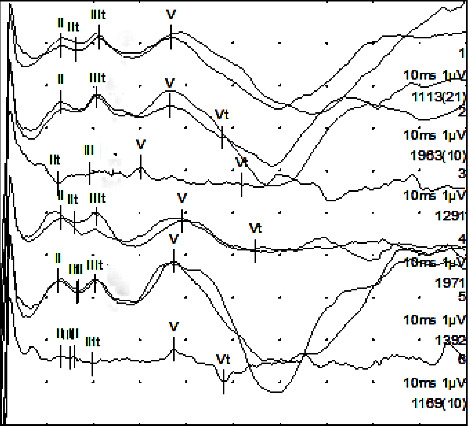
Recordings from the electric ABR response at different electrical stimulation intensities. The response showed bipolar stimulation between contacts 3 and 2 (apical contact and medial contact). It shows the presence of replicable waves III and V.

After inserting the electrode array, possible ABRs were detected on all three electrode contacts (waves II, III, and V), confirming the functionality of the auditory nerve (see Figure 6).

### 2.5. Intra- and Postoperative Imaging and Implant Functionality Testing

After confirming that it was possible to stimulate the auditory nerve in this case, the CI stimulator housing was placed in a bony bed under a subperiosteal pocket. The CI electrode array was fully inserted (until the marker) into the cochlea. Intraoperative radiography with Stenvers projection confirmed full insertion of the array into the cochlea, with an implantation depth reaching the second turn.

The telemetry of the intraoperative impedances showed impedance values within the normal range for all electrode contacts. AutoART (automated eCAP stimulation and recording) performed via the implant showed responses following stimulation with all intracochlear electrode contacts.

**Figure 7:**
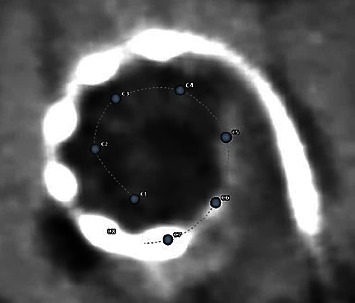
Implanted electrode array imaged via postoperative CT. The positions of the apical contacts are shown as numbers and dots after a 3D image reconstruction because not visible being on a different plane.

Postdischarge CT imaging, which was analysed using OTOPLAN, showed the insertion of the electrode array to an angular insertion depth of 644°, confirming the positioning of all the electrode contacts in the apical area of the cochlea as well as the ossified region (Figure 7).

### 2.6. Postoperative Treatment and Hearing Outcomes

Upon activation, all the intracochlear electrode contacts evoked the sensation of sound, with dynamic ranges similar to those observed in CI users implanted under more conventional conditions.

From the intracochlear electrode positioning determined from postoperative CT imaging and OTOPLAN analysis, the natural tonotopic frequency associated with each electrode contact position was estimated. This information was imported into the Maestro fitting software and used to assign frequency bands to the array contacts. This is an example of anatomy-based fitting, i.e., fitting of the CI which considers the patient's cochlear anatomical structure.

After approximately 2 years of CI use, the patient has a free-field PTA of 33 dB HL and a speech discrimination score of 90% at 70 dB HL (both with contralateral masking). For the Italian matrix sentence test in the S_0_N_0_ configuration, the SRT_50_ was +3.1 dB SNR.

No postoperative complications were reported.

## 3. Discussion

Temporal bone fracture is the cause of cochlear ossification in 9.5% of cases [9]. Cochlear ossification is still a challenge for the field, and multiple efforts are underway to overcome this challenge: the surgical technique (posterior tympanotomy, transcanal atticotomy, and subtotal petrosectomy), the choice of the electrode array (standard, compressed, and double array), the extent of the milling (round window, basal turn, middle turn, and drill out), and the location and depth of electrode array insertion (scala tympani or vestibular duct and complete or incomplete) in order to insert the maximum number of electrode contacts [9].

In the surgical planning for this case, we decided to perform a subtotal petrosectomy to ensure a broader approach oriented towards the cochlea [9, 15–17]. Electrode array selection was based on cochlear duct length, as determined with preoperative radiological analysis and the OTOPLAN software [11]. Furthermore, as it allows for the basal turn to be observed and to measure the extent of the ossification after the round window, this method enabled an electrode to be chosen, the active stimulation area of which could be inserted more apically. The goal of this approach was to bypass the ossified area of the cochlea and implant the array such that the electrodes were placed apically.

During the surgical intervention, having detected basal turn ossification, the technique used was to follow the direction of the basal turn of the scala tympani with the milling in order to obtain its patency from the round window; having not found the lumen, patency was found in the vestibular duct. No different outcomes are expected with this insertion method in comparison with the one in the scala tympani [9, 18–20].

The ANTS electrode test [19, 20] was used to test the residual functionality of the auditory nerve prior to inserting the electrode array of the CI. After inserting the electrode and performing the electrical stimulation via the Maestro software, potentials (eABRs) were detected on all three electrode contacts, confirming the functionality of the auditory nerve, which is a necessary condition for a good outcome with a CI. The CI electrode array was then fully inserted up until the marker. The intraoperative radiography with Stenvers projection confirmed the full insertion, showing the electrode array wrapped for around 2 turns.

Postoperatively, the patient's CI was programmed via anatomy-based fitting and, after 2 years, derives audiological benefit from CI use. No safety issues or complications have been reported.

## 4. Conclusion

From the experience of this clinical case study, the authors highlight how in complex cases such as cochlear ossification in temporal fractures with doubtful functionality of the cochlear nerve, the combination of (1) a broad surgical approach with a subtotal petrosectomy, (2) preoperative radiological assessment to measure cochlear duct length and thereby select the most appropriate electrode array length, and (3) the possibility of testing the residual functionality of the auditory nerve with the ANTS test prior to CI electrode array insertion, decreases risk of reduced benefits and promotes beneficial postoperative results.

## Data Availability

The image data used to support the findings of this study are included within the article.
